# Suboptimal human multisensory cue combination

**DOI:** 10.1038/s41598-018-37888-7

**Published:** 2019-03-26

**Authors:** Derek H. Arnold, Kirstie Petrie, Cailem Murray, Alan Johnston

**Affiliations:** 1 School of Psychology, The University of Queensland, St Lucia, Queensland 4102 Australia; 2 Experimental Psychology, University of Nottingham, Nottingham, UK

## Abstract

Information from different sensory modalities can interact, shaping what we think we have seen, heard, or otherwise perceived. Such interactions can enhance the precision of perceptual decisions, relative to those based on information from a single sensory modality. Several computational processes could account for such improvements. Slight improvements could arise if decisions are based on multiple independent sensory estimates, as opposed to just one. Still greater improvements could arise if initially independent estimates are summed to form a *single* integrated code. This hypothetical process has often been described as *optimal* when it results in bimodal performance consistent with a summation of unimodal estimates weighted in proportion to the precision of each initially independent sensory code. Here we examine cross-modal cue combination for audio-visual temporal rate and spatial location cues. While suggestive of a cross-modal encoding advantage, the degree of facilitation falls short of that predicted by a precision weighted summation process. These data accord with other published observations, and suggest that precision weighted combination is not a general property of human cross-modal perception.

## Introduction

Cues extracted from different sensory modalities can interact, bringing about striking changes in perception. In the spatial ventriloquist effect, for example, an auditory signal can seem to originate from the location of a visual signal^1^. This has been used by puppeteers to entertain for centuries, and it is pertinent whenever you watch television, and fail to notice that the visible actors aren’t actually making any sound, which is instead originating from an offset source. There is a related temporal effect. In temporal ventriloquism the timing of a visual event tends to be drawn toward that of an auditory signal^2^. As visual coding typically provides more precise information about the spatial environment, and auditory coding typically provides more precise timing information, these effects have historically been taken as evidence for the modality appropriateness hypothesis^3^ – the idea that a given sensory modality will dominate perception when that modality typically provides better information about the issue at hand.

In contemporary research, the modality appropriateness hypothesis has been overturned, due primarily to a seminal finding regarding the malleability of cross-modal perception. Instead of vision always dominating spatial perception, Alais & Burr^4^ showed that audition *could* dominate vision when the spatial cues provided by vision were sufficiently degraded. Instead of a certain type of sensory decision being dominated by a given sensory modality, researchers now argue that the brain estimates the instantaneous precision of each source of sensory evidence when multiple cues are available from different senses^4–8^. Hypothetically, the brain uses these estimates when it sums the initially independent sensory codes together to form an integrated code. This process is often referred to as an optimally weighted summation^5^.

A process of optimally weighted summation does not just allow for perceptual decisions to be dominated by diverse sensory modalities, it allows for enhanced sensitivity, relative to when information is available from just one sensory modality. This is true even if the two sensory modalities provide *equally* precise sensory estimates. Levels of sensitivity resulting from an optimally weighted summation can be predicted by employing a Minkowsky metric^9,10^ to evaluate the degree of summation. This can be calculated as follows:

1 $${\rm{AV}}s={({\rm{A}}{s}^{{\rm{k}}}+{\rm{V}}{s}^{{\rm{k}}})}^{1/{\rm{k}}}$$

where AV*s* denotes sensitivity to combined audio and visual signals, A*s* sensitivity to audio signals, and V*s* sensitivity to visual signals. The exponent, k, indicates the degree of summation, with an optimally weighted summation corresponding with a quadratic summation, or k = 2.

Note that in the cue combination literature the term “optimal” refers to an optimal combination rule under a given model. Since modeling efforts and experiments are not exhaustive, we can never say that a particular outcome is optimal in a general sense. Moreover, all such models are based on assumptions, so better or worse performance than model predictions can result if model assumptions are wrong^11^. Refuting model predictions does not, however, discredit the broader conceptual framework in which that model resides. Here that framework is Bayesian, with perception presumably informed by past contexts.

To avoid confusion, we will largely refrain from referring to optimal and suboptimal processes. Rather, we will examine the predictions of a specific model, wherein initially independent sensory estimates are combined via a process of weighted summation, with weights determined by an instantaneous and accurate appraisal of the precision of unimodal estimates. We will refer to precision weighted summation, as this is the rule many researchers have assumed^4,5,12^.

The provision of sensory cues in multiple, as opposed to just one, sensory modalities has repeatedly been found to result in more precise perceptual judgments^4,5,12–15^, and the degree of improvement has often been said to be consistent with a precision weighted summation^4,5,12,14^. But precision weighted summation is not the only combination rule that could result in bimodal improvements, and other schemes are seldom considered in detail, or pitted against precision weighted summation predictions in order to see which scheme best describes performance.

### Sensitivity differences predicted by different decision schemes

**Figure 1:**
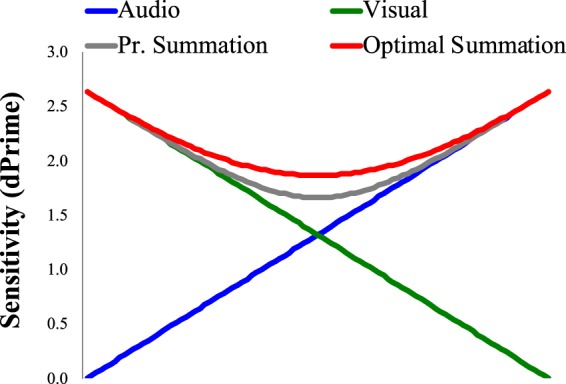
Plots of inversely varying simulated audio (blue) and visual (red) sensitivity scores, in addition to sensitivity scores predicted by weighted summations of unimodal signals (red) and via probability summations (grey). Note that the largest difference, between unimodal sensitivities and predicted bimodal sensitivities, occurs when unimodal sensitivities are *precisely* matched.

The idea that unimodal sensory estimates are combined via a precision weighted summation is conceptually important, and prominent in contemporary literature. However, the sensitivity differences this scheme predicts, relative to simpler decision schemes, are small. To illustrate, in Fig. 1 we show simulated unimodal (audio and visual) sensitivity scores (dPrime values) that vary inversely. We have also plotted bimodal sensitivities predicted assuming these signals are combined via a precision weighted summation process. Finally, we have plotted sensitivities predicted by assuming that each modality makes an independent contribution, on a trial-by-trial basis, to a decision process – probability summation^16,17^.

### Probability Summation

Pirenne^16^ pointed out that the ability to detect a signal could be enhanced if there were two encoded signals, with each having an independent probability (on a trial-by-trial basis) of exceeding an encoded level of intensity necessary for detection – a concept known as probability summation. The ability to detect audiovisual signals, at minimal component intensities, reportedly conforms to probability summation predictions^14,18^. The same logic applies to multisensory tasks – like deciding which of two presentations is associated with a greater value (further to the right, or higher in temporal modulation rate). If we assume the decision process weights information equally, a bimodal encoding advantage could still ensue, relative to even the most reliable single sensory modality, because either modality could provide a more intense correct signal on a trial-by-trial basis^17^. In terms of the Minkowsky metric we described earlier^9,10^, the difference between the predictions of a precision weighted summation, and a probability summation process, are encapsulated within the assumed exponent, k = 2 for precision weighted summations, 3 for probability summation.

### Comparing predicted bimodal sensitivities

The first thing to note about Fig. 1 is that the largest difference, between unimodal sensitivities and predicted bimodal sensitivities, happens when the two unimodal sensitivities are precisely matched. This is why multisensory cue combination studies often attempt to equate unimodal performances^4,5,14^. Note also that even these differences are slight. Unimodal dPrime values in this case are ~1.32, whereas bimodal sensitivity predicted by a precision weighted summation process is ~1.86. In a forced choice task, this could coincide with proportion correct scores of ~0.75 and 0.83 respectively. Any deviance from precisely matched unimodal sensitivities lessens the predicted advantage. For instance, if we assume a 5% difference in unimodal task performance, the better of the two modalities could coincide with ~0.77 correct task performance, whereas the predicted bimodal performance from precision weighted summation would still be ~0.83 – a 6% difference. Our point is that with any degree of measurement error, it might be difficult to distinguish between precision weighted summation predictions and decisions based on the best available unimodal evidence, and this constitutes a minimal condition before a multisensory decision process should be assumed.

Differences between bimodal sensitivities predicted by a precision weighted summation process, and those predicted by a probability summation process, are even smaller. If unimodal sensitivity dPrime scores are *precisely* matched (at 1.32), predicted dPrime values are 1.86 and 1.66 respectively, corresponding with proportion correct scores of ~0.83 and 0.80. Again, assuming any degree of measurement error, these slight predicted differences could be difficult to discern. Before any multimodal encoding advantage can confidently be attributed to a precision weighted summation process, probability summation should be dismissed as a viable interpretation, as this decision process predicts a bimodal advantage relative to averaged unimodal sensitivities, but it does not assume an analysis of the brains’ intrinsic signal to noise – an additional computation that is necessary for precision weighted summation calculations^4,5,12,14^.

### A minimal precision weighted summation prediction

Precision weighted summation predicts that sensitivity to a consistent combination of bimodal signals should be *greater* than sensitivity to the most precisely encoded unimodal signal, especially when unimodal sensitivities are matched (see Fig. 1). In our experiments we will use this as a metric to test if precision weighted summation predictions have been met, as this constitutes a minimal requirement before a multisensory process should be considered. We will also pit precision weighted summation predictions against probability summation predictions, to see which better describes performances on trials involving congruent bimodal signals.

To investigate these matters, we chose to examine audio, visual and audio-visual spatial origin (Experiments 1) and temporal rate (Experiment 2) judgments. To preface our results, we find evidence for enhanced audio-visual sensitivity in each context, relative to *average* unimodal performances. We do not, however, find that cross modal sensitivities are enhanced relative to the *most* precise unimodal sensitivity displayed by each participant. Also, in both experiments we find that probability summation better describes performance on congruent bimodal trials, relative to precision weighted summation predictions, although neither account accurately describes group-level performances. We also find that when audio and visual cues are placed in conflict, participant judgments are not biased in favor of the information that they had encoded with greater precision. All these results argue against bimodal decisions being informed by a weighted summation process.

## Experiment 1 – Audio, Visual and Audio-Visual Spatial Origin Judgments

### Methods

**Figure 2:**
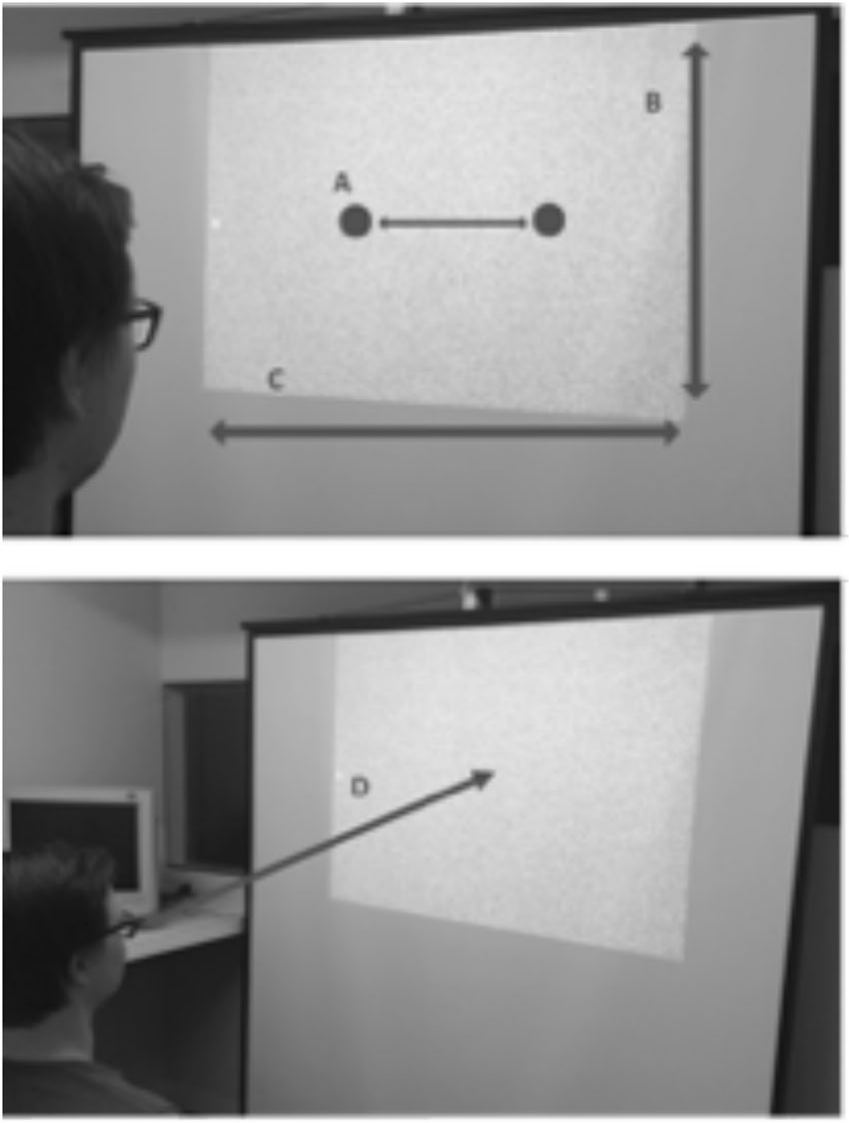
Graphics depicting the visual display for Experiment 1. (**A**) Position of speakers, mounted to the rear of the display, used to present audio stimuli, (**B**) Height of display area (86 cm), (**C**) Width of display area (112 cm) (**D**) Viewing distance (137 cm).

Ethical approval for both experiments was obtained from the University of Queensland Ethics Committee, and were in accordance with the Declaration of Helsinki. Consent to participate in the study was fully informed. Before each experimental session began, participants read an instruction screen that informed them that they could withdraw from the experiment at any time without penalty. They indicated consent to participate by clicking on a statement, that they had read and understood these instructions. The participant depicted in Fig. 2 consented to these images being used in an online open-access publication.

There were 20 participants in Experiment 1, including 2 of the authors and an additional 18 people who were naïve regarding the purpose of the experiment. Stimuli were generated using custom Matlab software in conjunction with the Psychophysics Toolbox^19,20^. Visual stimuli were presented via a NEC VT660 data projector. The data projector was positioned above and behind the participants’ head, 210 cm from the display screen (112 cm wide, 86 cm tall; see Fig. 2). Participants were seated in a chair, centered relative to the display screen, at a viewing distance of ~137 cm. Auditory stimuli were delivered via two speakers positioned 27 cm to the left and the right of the center of the display screen. These were mounted behind, centered on the vertical mid-point, and facing into the rear of the screen. Responses were reported by pressing one of two buttons on a mouse held in the participant’s lap.

Visual stimuli consisted of vertical Gabors, subtending ~15 degrees of visual angle (dva) at the retina, with a spatial constant of ~2.5dva, a spatial frequency of ~1 cycle per dva, and a Michelson contrast of 75%, shown against a grey background. These were flashed for 200 ms, followed by a full screen of visual white noise.

#### Audio calibration

Auditory stimuli consisted of white noise bursts, lasting for 25 ms with 5 ms linear onset/offset ramps. Eccentricity was signaled via inter-aural intensity differences. Before the experiment, participants completed a calibration task to determine what right speaker peak signal intensity was subjectively matched to a Standard left speaker peak intensity of ~60 dB SPL. In the subsequent experiment, apparent stimulus eccentricity was manipulated by presenting stimuli at multiples of these pre-determined peak stimulus intensities, such that a signal apparently originating 27 cm to the *left* of the display center would have a peak left channel intensity of ~60 dB SPL while the right channel was silent, and a signal apparently originating from the display center was signaled by both channels being set to half their pre-determined peak intensities.

#### Performance calibration

A second calibration phase was used to identify signaled eccentricities for unimodal stimulus presentations resulting in ~67% correct task performance. Subjectively, Standard signals seemed to originate from the display center, whereas Comparison signals were manipulated on a trial-by-trial basis to identify signaled eccentricities that could be accurately identified, as originating from the left or right relative to Standard presentations on ~67% of trials. During this procedure signaled Comparison eccentricities were adjusted according to 1-Up, 2-Down staircase procedures, wherein an incorrect judgment resulted in an increased Comparison eccentricity, and two consecutive correct judgments in a decrease in Comparison eccentricity being signaled. Audio and Visual Comparison stimuli for the subsequent experiment were set to these values, to ensure performance would be closely matched across unimodal stimulus conditions. This level of task performance was chosen to avoid floor and ceiling effects in the subsequent experiment.

#### Test Phase

Each trial consisted of two sequential presentations, of a Standard then a Comparison, or of a Comparison then a Standard stimulus (order determined at random on a trial-by-trial basis). Stimulus presentations were separated by a 333 ms inter-stimulus interval (ISI). On each trial the participant was required to indicate whether the second stimulus presentation was offset to the left or right relative to the first. The direction of the Comparison offset (left/right) was determined at random on a trial-by-trial basis.

Four types of trial were interspersed during trial blocks. Stimulus presentations were either auditory, visual, congruent audiovisual, or incongruent audiovisual. In congruent audiovisual trials, the Comparison offset direction (left or right) signaled by audition and vision was matched, whereas in incongruent audiovisual trials, audition and vision signaled *opposite* offset directions. Each trial block consisted of 80 individual trials for each type of trial, 320 individual trials in total, all interspersed in random order. Each participant completed three blocks of trials, and data were collated across the three blocks prior to analysis, for a total of 960 individual trials for each participant.

## Results

Uunimodal performance levels were well matched, both across individuals (visual 65%, audio = 64%) and within individuals (mean individual difference score of 0.7% SD = 4.8).

**Figure 3:**
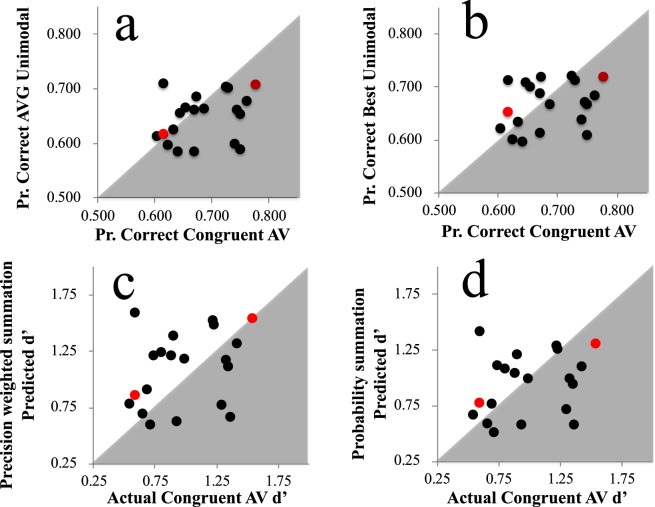
(**a**) X/Y scatterplot of individual proportion correct task performance scores on Congruent audiovisual trials (X axis) and averaged across unimodal visual and auditory trials (Y axis). (**b**) As for (**a**), but for the best of each individuals’ unimodal condition performance (Y axis). (**c**) X/Y scatterplot of individual d’ scores for Congruent audiovisual trials (X axis) and d’ scores predicted by precision weighted summation, from performances on unimodal trials. (**d**) As for (**c**), but for d’ scores predicted by probability summation. In all plots author data points are coloured red. Data points in grey regions of (**a**,**b**) indicate *better* performance on Congruent AV trials, relative to the other dataset. Data points in white regions in (**c**,**d**) indicate *worse* performance on congruent AV trials than predicted.

Of our participants, 15 of 20 showed *some* evidence of a bimodal encoding advantage, with performance on congruent audiovisual trials *superior* to performances averaged across each participants’ two unimodal conditions (see Fig. 3a). Averaged across participants, congruent audiovisual performance (69% SD = 6) was *better* than averaged unimodal performances (65% SD 4; paired t_19_ = 2.96, *p* = 0.008, 95% CIs 0.012 to 0.068). Congruent audiovisual performance was not, however, improved relative to the best unimodal performance achieved by each participant (67% SD 4; paired t_19_ = 1.48, *p* = 0.155, 95% CIs −0.008 to 0.049; see Fig. 3b).

Proportion correct scores for audio, visual, and congruent AV trials were converted into hit rates and false alarm rates, by respectively treating left and right offset cues as ‘signal’ and ‘noise’. This allowed us to calculate d’ scores for congruent AV trials, and predicted d’ scores based on unimodal trials (following the formula outlined in the introduction). Predictions for precision weighted summation and probability summation are plotted against actual congruent AV d’ scores in Fig. 3c,d respectively. Of the two schemes, probability summation predictions were *closer* to actual congruent AV d’ scores. Each set of predictions were subjected to Bayes factor analysis for a paired samples t-test, using JASP software (2015) with a Cauchy prior width of 0.707. These compared model predictions to actual performances on congruent bimodal trials. For precision weighted summation predictions, this only revealed anecdotal evidence in favour of the null hypothesis (that model predictions would be equivalent to actual bimodal performance; BF_10_ = 0.418, error 0.015%). For probability summation predictions there was moderate evidence in favour of the null hypothesis (BF_10_ = 0.233, error 0.022%).

### Incongruent audio-visual trial bias

**Figure 4:**
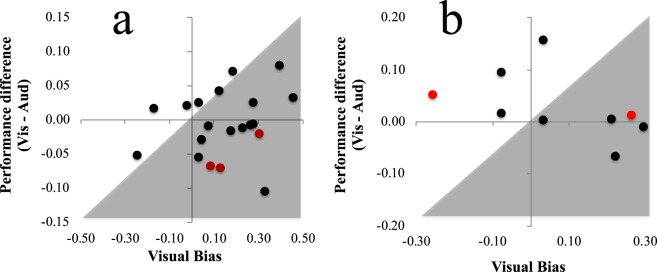
(**a**) X/Y scatterplot of individual visual bias scores (X axis) and proportional differences in correct performances on unimodal visual and audio trials (Y axis) from Experiment 1. Data points falling within the grey region are indicative of a greater visual bias than is justified by differences in unimodal trial performances. (**b**) Details are as for (**a**) but data are from Experiment 2.

As unimodal performance levels were well matched on average, if people had accurate insight into the precision of information provided by audition and vision, there should have been no systematic group-level bias on Incongruent audiovisual trials (when unimodal spatial cues were placed in conflict). This was tested by calculating individual bias scores, by subtracting the proportion of incongruent audiovisual trials on which a participants’ response was consistent with the visual stimulus, from the proportion of such trials on which their response had been consistent with the audio stimulus. A positive bias score is indicative of a visual bias, a negative score an audio bias. Averaged across participants, we found evidence for a visual bias (15% SD = 18, single sample t_19_ = 3.81, *p = *0.001, 95% CIs 0.069 to 0.236, see Fig. 4a). This provides further evidence that bimodal decisions lack insight into how well information in either uni-sensory modality has been encoded. If they had, bias scores should have reflected the modality that had prompted better unimodal performance, but there was no robust correlation between individual performance levels on unimodal trials and the degree to which people displayed a visual bias on incongrunet bimodal trials (Pearson’s R = 0.2, p = 0.388, 95% CIs −0.26 to 0.59).

## Discussion

We found evidence for a bimodal encoding advantage, in that individual performance tended to be *enhanced* on Congruent audiovisual trials relative to each individuals’ performance averaged across unimodal (audio *and* visual) trials. Congruent audiovisual trial performance was, however, inferior on average to each individuals’ performance on the *best* of their two unimodal conditions. Moreover, performance on Incongruent audiovisual trials suggested that participants had placed an undue emphasis on visual cues in those trials, as participants had displayed a visual bias despite the two unimodal cues being well-matched in terms of task performance. This speaks against bimodal decisions being guided by an *accurate* sensory-level appraisal of the precision of unimodal cue encodings, as predicted by a precision weighted summation process^4,5,12^.

Of the two bimodal decision schemes we have outlined (probability summation and precision weighted summation), probability summation predictions were more accurate (see Fig. 2c,d). Neither scheme, however, predicted the data well. This is not surprising in light of the superiority of each participants’ best unimodal performance, relative to performances on congruent bimodal trials. This implies that participants based decisions predominantly on one type of unimodal information on congruent bimodal trials, and that this was not reliably the best source of information available to them (so they did not benefit from an integrative process). In Experiment 2 we decided to see if a similar pattern of results would emerge when people make temporal judgments.

## Experiment 2

### Methods

There were 9 participants, including 2 of the authors and 7 people who were naïve as to the purpose of the experiments.

Both visual and auditory signals were generated using a TDT (Tucker-Davis Technologies) psychoacoustic workstation, to drive a green light-emitting diode (LED) mounted directly in front of a speaker used to present sounds. Presentations persisted for 0.75 to 1.5 seconds (10 ms linear onset and offset ramps), with precise presentation duration determined at random on a presentation-by-presentation basis. Randomising presentation times was necessary to avoid event counting from being a reliable cue as to modulation rate. All trials involved a sequential presentation of a Standard signal (sinusoidally modulated at 10 Hz) and a Comparison (modulated at a slower or faster rate), separated by an inter-stimulus-interval of between 0.5 and 1.25 seconds. Presentation order was randomized on a trial-by-trial basis. Participants were required to indicate which interval had contained the faster modulation rate, by pressing one of two mouse buttons.

### Performance calibrations

People tend to be more sensitive to temporal modulations signaled by audition relative to vision. All participants therefore completed a two-stage preliminary calibration process, first to identify slower and faster visual Comparison rates, than a 10 Hz Standard, that could be detected on ~70% of trials. We then determined what intensity of non-modulated audio noise needed to be added to sinusoidally-modulated audio noise signals to reduce audio performance to the same level (~70% correct) as visual performance.

### Visual calibration

Visual signals were generated by sinusoidally modulating the LED (peak intensity ~125 cd/m^2^). Standard signals were modulated at 10 Hz. During blocks of trials Comparison rates were modulated (in 0.25 Hz steps) according to 1-up 2-down staircase procedures, wherein incorrect judgments resulted in Comparison rates becoming more dissimilar to 10 Hz Standards, and two sequential correct answers in Comparison rates becoming more similar. Staircase procedures were instigated at Comparison rates that differed from 10 Hz Standards by 2.5 Hz (so 7.5 Hz for slower Comparisons, and 12.5 Hz for faster comparisons). A minimal difference of 0.25 Hz was enforced for both staircases.

A block of trials consisted of 80 individual trials, 40 for slower Comparisons, and 40 for faster Comparisons, all interleaved in random order. This provided distributions of correct rate discrimination performance as a function of Comparison rates. We fit logistic functions to these data, and took 75% points on fitted functions as an estimate of detectable Comparison rates.

### Audio calibration

Slower and faster Comparison rates were set to the detectable Comparison rates determined by the visual calibration procedure. The peak intensity of sinusoidally modulated audio white noise was fixed (peak intensity ∼63 dB SPL). Unmodulated audio white noise signals were added to limit performance. During a block of trials the peak intensity of non-modulated audio white noise was adjusted according to a 1-up 2-down staircase procedure, wherein incorrect responses resulted in a decrease in amplitude and 2 successive incorrect responses in an increase in amplitude. The staircase procedure was instigated at a peak intensity of 0 dB SPL, and adjusted in steps of 6.3 dB SPL until the first incorrect response, and then by 3.15 dB SPL). A block of trials consisted of 120 individual trials, 60 for slower Comparisons and 60 for faster Comparisons.

Trial blocks provided distributions describing the proportion of correct rate discriminations as a function of unmodulated audio white noise intensity. We fit logistic functions to these data, and took 75% points on fitted functions as an estimate of the requisite level of unmodulated audio white noise to equate audio and visual rate discrimination performance for that participant.

### Test Phase

Comparison type (slower/faster) was determined at random on a trial-by-trial basis. Four trial types were interspersed during trial blocks. Stimulus presentations were either auditory, visual, congruent audiovisual, or incongruent audiovisual. In congruent audiovisual trials, audio and visual Comparisons were matched (both slower or both faster) and were presented in-phase with one another. In incongruent audiovisual trials, audio and visual Comparisons were *opposite* (one slower the other faster). Each trial block consisted of 30 individual trials for each type of trial, 120 individual trials in total, interspersed in random order. Each participant completed three blocks of trials, and data were collated across the three blocks prior to analysis, for a total of 360 individual trials for each participant.

## Results

Uunimodal performance levels were well matched, both across individuals (visual 73% SD 7, audio = 70% SD 6) and within individuals (mean individual difference score 3% SD = 6, paired t_8_ = 1.35, p = 0.21, 95% CIs −0.02 to 0.08).

**Figure 5:**
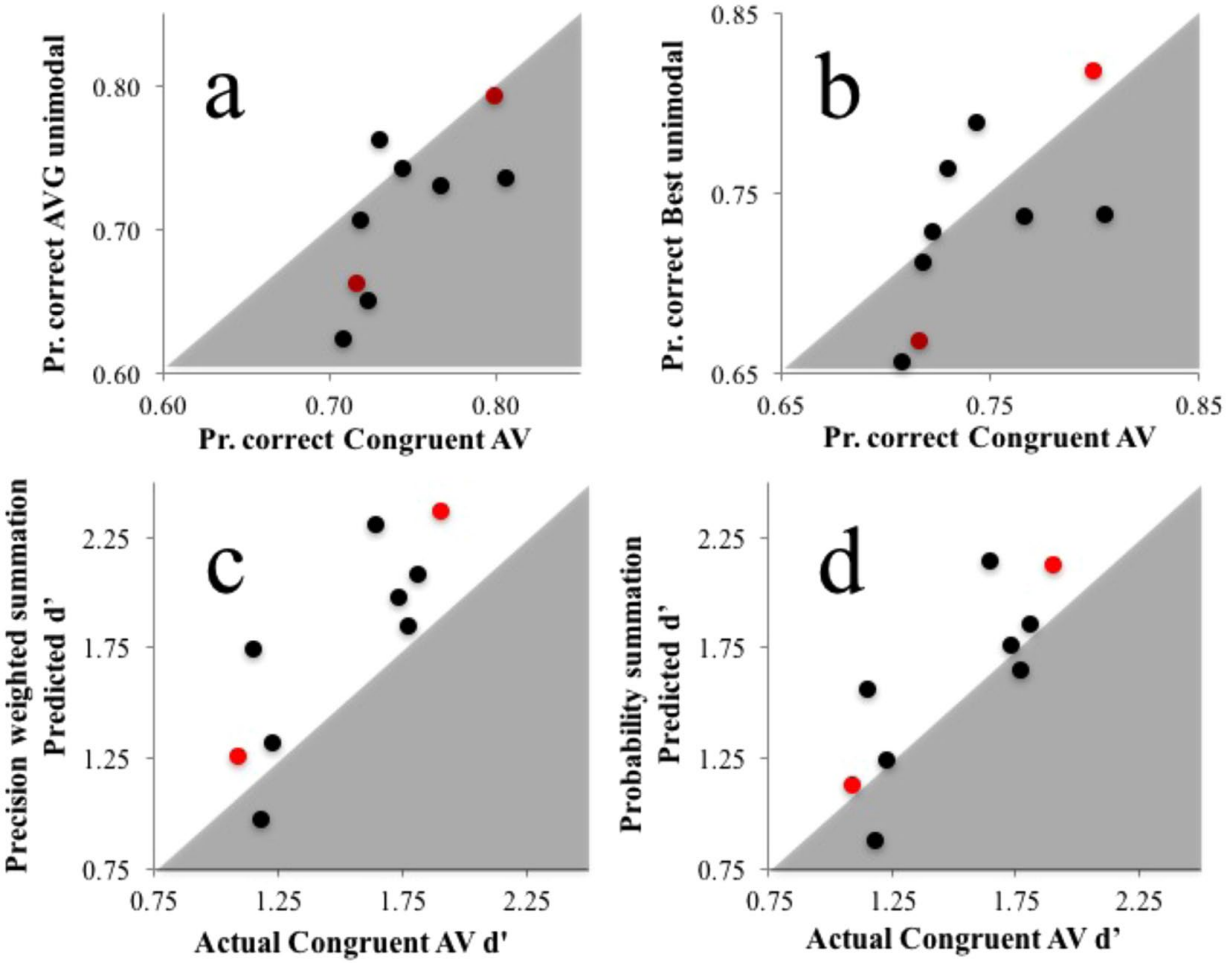
Data from Experiment 2. (**a**) X/Y scatterplot of individual proportion correct task performance scores on Congruent AV trials (X axis) and proportion correct scores averaged across each individuals’ unimodal visual and auditory trials (Y axis). (**b**) As for (**a**) but for the best of each individuals’ two types of unimodal trials (Y axis). (**c)** (**c**) X/Y scatterplot of individual d’ scores for Congruent AV trials (X axis) and d’ scores predicted by a precision weighted summation, from performances on unimodal trials. (**d**) As for (**c)**, but for d’ scores predicted by probability summation. In all plots author data points are coloured red. Data points in grey regions of (**a**,**b**) indicate *better* performance on Congruent AV trials, relative to the other dataset. Data points in white regions in (**c**,**d**) indicate *worse* performance on congruent AV trials than predicted.

Of our participants, 8 of 9 showed *some* evidence of a bimodal encoding advantage, with performance on congruent audiovisual trials superior to performances averaged across each individuals’ two unimodal conditions (see Fig. 5a). Averaged across participants, congruent audiovisual performance (75% SD = 4) was *better* than averaged unimodal performances (71% SD 6; paired t_8_ = 2.7, *p* = 0.027, CIs 0.005 to 0.066). Congruent audiovisual performance was *not*, however, improved relative to the best unimodal performance achieved by each participant (73% SD 5; paired t_8_ = 0.85, *p* = 0.419, CIs −0.02 to 0.04; see Fig. 5b).

As in Experiment 1, we converted proportion correct scores for audio, visual, and congruent AV trials into hit rates and false alarm rates, as per signal detection theory, by treating faster and slower test cues as ‘signal’ and ‘noise’ respectively. We then calculated d’ scores for audio, visual and congruent AV trials, and predicted d’ scores based on unimodal trials. Predictions for precision weighted summation and probability summation are plotted against actual congruent AV d’ scores in Fig. 5c,d respectively. Of the two schemes, probability summation predictions were closer to actual congruent AV d’ scores. Again, each set of predictions were subjected to Bayesian factor analyses for paired samples t-tests, using JASP software (2015) with a Cauchy prior width of 0.707. For precision weighted summation predictions, this only revealed moderate evidence in favour of the *alternate* hypothesis (that model predictions would differ from actual performance on congruent bimodal trials; BF_10_ = 3.098, error 0.001%). For probability summation predictions, however, this revealed anecdotal evidence in favour of the null hypothesis (that model predictions would be equivalent to actual performance on congruent bimodal trials, BF_10_ = 0.474, error 0.016%).

### Incongruent audio-visual trial bias

As in Experiment 1, as unimodal performance levels were well matched, if people had accurate insight into the precision of information provided by audition and vision, there should have been no systematic bias on Incongruent audiovisual trials. So, as in Experiment 1, we calculated individual modality bias scores, by subtracting the proportion of incongruent audiovisual trials on which a participants’ response was consistent with the audito stimulus, from the proportion of such trials on which their response was consistent with the visual stimulus. Averaged across participants, there was a trend toward a positive visual bias (7% SD = 19), but this was not statistically significant (single sample t_8_ = 1.12, *p* = 0 0.297, 95% CIs −0.08 to 0.22, see Fig. 4b).

## Discussion

Overall, we found that audiovisual performance was *superior* to performance averaged across unimodal (audio and visual) trials. These data are generally consistent with previous reports of cross-modal encoding advantages^5,12,13^, and specifically with bimodal audio-visual improvements in sensitivity to spatial position^4^ and temporal modulation rates^14,15^. The advantages we observed were, however, slight and did not reach precision weighted summation predictions in either experiment.

Our data stand in conflict with suggestions that unimodal sensory estimates are combined via a precision weighted summation process^4,5,12^. Of the two bimodal decision schemes we described in the introduction (probability summation and precision weighted summation), performance on congruent AV trials in both experiments was better described by a probability summation, wherein people base decisions on the strongest unimodal signal encoded on each trial (compare Figs 3c with 3d, and 5c with 5f). Unlike precision weighted summation, this scheme does not presume that the brain has insight into its own encoding precisions; decisions are just guided by the strongest encoded signal. With that said, neither scheme was a good predictor of performance on congruent AV trials. This is unsurprising, given that performance on these trials fell short of the best performance achieved by each participant for a single sensory modality. Overall, therefore, there was no evidence for a bimodal encoding advantage. Participants would have performed better in both experiments if they had been able to identify which sensory modality had provided slightly better information to them on average, so they could rely on this information exclusively on congruent bimodal trials.

### Is there a better account of bimodal perceptual decisions?

While our data show that probability summation better describes the performance of our participants on congruent bimodal trials, relative to weighted summation, we emphasize that neither account provided a very good description of our data (see Figs 3c,d and 5c,d). The lack of clustering, along a slope describing performance on congruent bimodal trials in relation to either set of model predictions, suggests to us that accurate performance descriptions will ultimately require a number of accounts, with different people adopting different decision rules when confronted with multiple sensory cues^21^. This would explain why some people benefit substantially from congruent bimodal presentations, whereas others are disrupted, and would do better if they could identify which sensory modality had provided better information on average. We acknowledge, however, that a single computational model could be devised that provides a comprehensive account of multi-sensory decision making, but suspect it would have to have multiple possible states to describe different peoples’ decisions. These multiple states/parameter settings could reasonably be described as distinct accounts of the decision process. Our main point for now is that none of the currently popular accounts of multisensory decision making provides a good comprehensive description of our data.

### Visual Bias

Precision weighted summation outcomes can only be achieved if evidence is *accurately* weighted, in proportion to the variance associated with each contributing sensory estimate. Any bias on incongruent bimodal trials should reflect these weightings, with decisions biased in favour of the information that is slightly more precise for that participant. Perusal of Figs 4 and 5 show that this did not happen in our experiments. In Experiment 1 participants relied primarily on visual information, with 18 of 20 participants biased to make reports consistent with visual signals, despite unimodal sensitivities being well matched (see Fig. 4). Biases in Experiment 2 were not overall consistent, but nor did they accurately reflect the more precise information for each participant (see Fig. 5). Experiment 1 echoes the modality appropriateness hypothesis, as vision typically provides more accurate spatial location cues^3^. In that experiment our participants might have been unable to ignore a life-time of experience, precluding the possibility of obtaining an advantage from having an additional sensory cue, even if that cue was encoded more precisely.

The lack of correlation, between visual biases on incongruent bimodal trials and differences in unimodal performance levels, does not dictate that congruent and incongruent bimodal trials must have been informed by distinct computational processes. That need only follow if you assume that performance on congruent bimodal trials is informed by a process that has insight into how well unimodal signals have been encoded. Our data do not provide any evidence in favour of this. Instead of assuming that congruent and incongruent trials reflect distinct computational processes, one can assume that both sets of decision are informed by a process that lacks accurate information into how well information has been encoded in either modality.

We did not quiz people on their awareness that sensory cues were sometimes in conflict, although anecdotally this was not readily apparent. Slight perceptual differences, balanced in terms of encoded magnitude, can sum to form sensory metamers, which are indistinguishable from consistent cues signaling an average sensory magnitude^12^. Our cues were calibrated, such that in isolation they were encoded with equal *modest* accuracy (prompting correct performance on ~70% of trials). These cues would therefore often have been experienced as a sensory metamer, promoting perception of the average sensory value.

Asking participants about cue conflict during an experiment could encourage people to ignore one or the other sensory modality on bimodal trials, whereas we wanted them to attend both. If participants detected conflict, and opted to ignore either sensory modality of their own accord, this would speak against decisions being informed by an *accurate* automated sensory-level weighting process. Such a process should have led to a bimodal encoding advantage when cues were consistent, and to sensory modalities being relied on equally (at the group-level) on conflicting bimodal trials (as each modality in each experiment was associated with equal precision). One could argue that a disproportionate reliance on visual information makes sense if conflict is detected, due to life experience, resulting in a systematic bias and a failure to benefit from redundant cues on consistent bimodal trials. This again, however, would be inconsistent with an automated sensory-level weighting of information, as predicted by precision weighted summation^4,5,12,22^.

### Why no feedback?

Readers should note that we did not provide participants with feedback re task performance. First, this was because we did not want to disrupt any automated sensory-level processes, by providing information that could prompt a cognitive re-appraisal of response strategy. Other researchers have suggested that the human brain encapsulates sensory-level processes, that measure the precision of unimodal sources of information, so that they can be appropriately weighted in decision processes^4,5,12,22^. Providing feedback could have disrupted this operation. A second reason is that feedback becomes meaningless when equally precise cues are in conflict.

### How do our findings relate to existent literature?

There is now a reasonably large literature concerning bimodal sensitivity improvements. Numerous findings are said to be well predicted by a precision weighted summation process^4,5,23^, but not all^6,24–26^. When it comes to sensitivity, we note that predictions generally *are not* compared to probability summation, but rather to either average unimodal performances^6^ or sometimes to the best unimodal performance achieved by each participant^27^. Probability summation and optimally weighted summation make very similar predictions regarding bimodal sensitivity improvements, but only optimally weighted summation assumes accurate insight into the precision with which unimodal signals have been encoded by the brain. Thus, unless evidence dictates otherwise, we feel that parsimony demands that probability summation should be preferred as an explanation. We hasten to point out, however, that neither account provided a very compelling account of our data.

We shall briefly consider two prominent studies to illustrate these issues. Alais & Burr^4^ tested 3 and 6 participants in two experiments, closely matched to our Experiment 1. In the first the most experienced observer displayed evidence for a bimodal encoding advantage consistent with precision weighted summation predictions, whereas the other two participants displayed lesser advantages. In the second all 6 participants were more accurate when localizing congruent bimodal signals, relative to unimodal signals. For 2 of 6 participants, bimodal performance exceeded precision weighted summation predictions, but this was reversed for the other 4. As a group, average performance was well-described by precision weighted summation, but precision weighted summation predictions were not pitted against probability summation – which predicts a similar benefit but assumes less computations (see Fig. 1). The authors did, however, show that audition could dominate a sufficiently degraded visual signal when the two were placed in conflict, which challenges the modality appropriateness hypothesis. Overall, these authors drew an appropriately cautious, but influential, conclusion – that bimodal performance had been *consistent* with a *near* optimal bimodal integration process.

Ernst & Banks^5^ had four participants judge the height of bars, signaled either by touch or sight. They added noise to visual signals to vary the reliabilities of the two cues. They found that bimodal performance, averaged across participants, approximated the level of precision predicted by a precision weighted summation. However, of the four noise conditions only one was characterized by bimodal performance that was *clearly* distinguished from that promoted by the best unimodal cue, and precision weighted summation predictions were not pitted against probability summation.

In sum, these two prominent papers did not compare the precision weighted summation model against a simpler unimodal decision process, and thus they do not provide a basis for discounting the simpler model.

### Implications

The clearest implication of our data is that bimodal sensory decisions are not *generally* based on a precision weighted summation process. This, of course, does not dictate that this is either impossible, or that it never happens. One possibility is that bimodal integration processes were not triggered by our experiments. The spatial cues in Experiment 1 were perhaps insufficiently matched, and temporal rate modulations may be encountered so rarely in daily life that they fail to trigger precision weighted summation processes. How, then, could we tell, in different circumstances, if a precision weighted summation process had been triggered? Perhaps the simplest way would be to have a sufficiently powered study to distinguish between the very slight sensitivity differences predicted by precision weighted summation and by probability summation. This, however, might seldom be practical.

Some previous studies have manipulated unimodal sensitivities, by adding noise or by relatively degrading a contributing signal, in order to induce a systematic bias in favour of the more precise signal. The success of these manipulations has also been taken as evidence for precision weighted summation process^4,5^. But these results are potentially consistent with the predictions of probability summation. According to an evenly weighted probability summation, the decision acts on the strongest pertinent signal on a trial-by-trial basis. The addition of noise that makes it harder to extract a pertinent signal could lessen the strength of evidence available to the decision process, resulting in decisions being based on the stronger source of evidence. In the extreme, trials wherein a degraded unimodal signal had exceeded an intensity level necessary to reach a decision might be interspersed with trials where signals had not, resulting in guessing in the absence of another cue. We therefore believe a more convincing demonstration of precision weighted summation predictions would be to examine signals that are closely matched, in terms of resulting behavioural sensitivity, and examine if individual biases scale with slight individual differences in unimodal sensitivities – as we have done. This, in combination with a demonstration that sensitivity improvements *exceed* the predictions of an evenly weighted (probability) summation, would be conclusive. Neither finding is apparent in our data.

It has been argued that precision weighted summation predictions might exceed actual performance if the process of estimating cue weights is subject to noise^4^. From an empirical perspective, this would be problematic – potentially making the hypothesis unfalsifiable.

### Why do we care?

If unimodal cues are summed in proportion to the precision with which they have been encoded, we need to consider how this might be achieved by the nervous system. Candidate proposals have been put forward^22,28^. It is unclear to us, however, if any such proposal is necessary.

### What we have and have NOT done

Bayesian inference is growing in popularity as an account of sensory processing in general, and of human vision in particular^29^. Broadly speaking, this framework assumes that perception results from evolutionary adaptations that have a specified function, and that evolution has arrived at the optimal means of fulfilling these functions given prevailing constraints. If researchers correctly guess what evolutionary function a process fulfills, and what constraints that process is subject to, they can construct a model that predicts optimal performance^11^. All that can be said of any particular Bayesian inspired model is that it is a good or bad predictor of human behavior. If it is a poor predictor (like precision weighted summation in our experiments), the pertinent model may have misconstrued the evolutionary function, or it may not be fully informed of contextual constraints. Finding that a particular model is a poor descriptor does not, however, refute the Bayesian framework as an account of brain function per se, it just highlights the inadequacy of a particular model.

### Take Home Message

Our data show that bimodal decision-making does not always result from a precision weighted summation of unimodal sensory cues – a possibility often described as ‘optimal integration’. Bimodal advantages fell short of precision weighted summation predictions, and were better described by probability summation, which does not assume an instantaneous assessment of the precision with which the brain has encoded unimodal information^17,22,28,29^. We also point out that some of the evidence advanced in favour of precision weighted summation is inconclusive, and that the predictions of this scheme are seldom compared to other schemes that assume less computational insight (the precision with which the brain has encoded unimodal sensory information)^4–6,27^. We do not assert that our alternate model (probability summation) provides a very good account of bimodal decisions – just a better description than precision weighted summation (which is overwhelmingly assumed in contemporary research). So our final take home message is that, far from being settled, the processes by which sensory cues are combined require further clarification – these are not generally mediated by a precision weighted summation process.

## References

[1] Slutsky DA, Recanzone GH (2001). Temporal and spatial dependency of the ventriloquism effect. NeuroReport.

[2] Vroomen J, de Gelder B (2004). Temporal ventriloquism: Sound modulates the flash-lag effect. Journal of Experimental Psychology: Human Perception and Performance.

[3] Welch RB, Warren DH (1980). Immediate perceptual response to intersensory discrepancy. Psychological Bulletin.

[4] Alais D, Burr D (2004). The Ventriloquist effect results from near optimal bimodal integration. Current Biology.

[5] Ernst MO, Banks MS (2002). Humans integrate visual and haptic information in a statistically optimal fashion. Nature.

[6] Roach NW, Heron J, McGraw PV (2006). Resolving multisensory conflict: A strategy for balancing the costs and benefits of audio-visual integration. Proceedings: Biological Sciences.

[7] Van Beers RJ, Sitting AC, Gon JJ (1999). Integration of proprioceptive and visual position-informaion: An experimentally supported model. Journal of Neurophysiology.

[8] Van Dam, L. C. J., Parise, C. V., & Ernst, M. O. Modeling multisensory integration. In book: *Sensory integration and the unity of consciousness*. MIT Press, CT: 209–229 (2014).

[9] Meese TS, Harris MG (2001). Independent detectors for expansion and rotation, and for orthogonal components of deformation. Perception.

[10] Meyer GF, Wuerger SM, Rohrbein F, Zetzsche C (2005). Low-level interation of auditory and visual motion signals requires spatial co-localisation. Experimental Brain Research.

[11] Anderson B (2015). Can Computational Goals Inform Theories of Vision?. Topics in Cognitive Science.

[12] Hillis JM, Ernst MO, Banks MS, Landy MS (2002). Combining sensory information: Mandatory fusion within, but not between, senses. Science.

[13] Arnold DH, Tear M, Schindel R, Roseboom W (2010). Audio-Visual Speech Cue Combination. PLoS One.

[14] Koene. A., Arnold, D. H. & Johnston, A. Bimodal sensory discrimination is finer than dual single modality discrimination. *Journal of Vision*, **7**(11): 14, 1–11 (2007). 10.1167/7.11.14 17997668

[15] Vroomen J, de Gelder B (2000). Sound enhances visual perception: Cross-modal effects of auditory organization on vision. Journal of Experimental Psychology: Human Perception and Performance.

[16] Pirenne MH (1943). Binocular and uniocular threshold of vision. Nature.

[17] Treisman A (1998). Feature binding, attention and object perception. Phil Trans R Soc Lond B.

[18] Mulligan, Shaw RM, Shaw ML (1980). Multimodal signal detection: Independent decision vs integration. Perception & Psychophysics.

[19] Brainard D (1997). The psychophysics toolbox. Spat Vis.

[20] Kleiner M (2007). What’s new in Pyschtoolbox-3. Perception.

[21] Ball DM, Arnold DH (2017). and Yarrow, K. Weighted integration suggests that visual and tactile signals provide independent estimates about duration. Journal of Experimental Psychology: Human Perception and Performance.

[22] Fetsch CR, Pouget A, DeAngelis GC, Angelaki DE (2012). Neural correlates of reliability-based cue weighting during multisensory integration. Nature Neuroscience.

[23] Helbig HB, Ernst MO (2007). Optimal integration of shape information from vision and touch. Exp Brain Res.

[24] Battaglia PW, Jacobs RA, Aslin RN (2003). Bayesian integration of visual and auditory signals for spatial localization. J Opt Soc Am A.

[25] Lukas S, Philipp AM, Koch I (2014). Crossmodal attention switching: Auditory dominance in temporal discrimination tasks. Acta Psychologia.

[26] Wada Y, Kitagawa N, Noguchi K (2003). Audio-visual integration in temporal perception. Int J Psychophysiol.

[27] Oshiro T, Angelaki DE, DeAngelis GC (2011). A normalization model of multisensory integration. Nature Neuroscience.

[28] Stekelenburg JJ, Vroomen J (2007). Neural correlates of multisensory integration of ecologically valid audiovisual events. Journal of Cognitive Neuroscience.

[29] Knill D, Pouget A (2004). The Bayesian brain: the role of uncertainty in neural coding and computation. Trends in Neurosciences.

